# Wafer‐Level Manufacturing of MEMS H_2_ Sensing Chips Based on Pd Nanoparticles Modified SnO_2_ Film Patterns

**DOI:** 10.1002/advs.202302614

**Published:** 2023-07-03

**Authors:** Zheng Zhang, Liyang Luo, Yanlin Zhang, Guoliang Lv, Yuanyuan Luo, Guotao Duan

**Affiliations:** ^1^ School of Integrated Circuits Huazhong University of Science and Technology Wuhan 430074 China; ^2^ Key Laboratory of Materials Physics Institute of Solid State Physics HFIPS Chinese Academy of Sciences Hefei 230031 China; ^3^ Wuhan National Laboratory for Optoelectronics Huazhong University of Science and Technology Wuhan 430074 China

**Keywords:** film patterns, H_2_ sensing chips, high‐consistency, micro‐electro‐mechanical systems, wafer‐level

## Abstract

In this manuscript, a simple method combining atomic layer deposition and magnetron sputtering is developed to fabricate high‐performance Pd/SnO_2_ film patterns applied for micro‐electro‐mechanical systems (MEMS) H_2_ sensing chips. SnO_2_ film is first accurately deposited in the central areas of MEMS micro hotplate arrays by a mask‐assistant method, leading the patterns with wafer‐level high consistency in thickness. The grain size and density of Pd nanoparticles modified on the surface of the SnO_2_ film are further regulated to obtain an optimized sensing performance. The resulting MEMS H_2_ sensing chips show a wide detection range from 0.5 to 500 ppm, high resolution, and good repeatability. Based on the experiments and density functional theory calculations, a sensing enhancement mechanism is also proposed: a certain amount of Pd nanoparticles modified on the SnO_2_ surface could bring stronger H_2_ adsorption followed by dissociation, diffusion, and reaction with surface adsorbed oxygen species. Obviously, the method provided here is quite simple and effective for the manufacturing of MEMS H_2_ sensing chips with high consistency and optimized performance, which may also find broad applications in other MEMS chip technologies.

## Graphical abstract

A simple method combining atomic layer deposition and magnetron sputtering is reported to fabricate high‐performance Pd/SnO_2_ film patterns for H_2_ sensing, which can be effectively used for wafer‐level fabrication of micro‐electro‐mechanical systems (MEMS) gas sensing chips or other MEMS chips. The H_2_ sensing chips show a wide detection range from 0.5 to 500 ppm, high resolution, and good repeatability.

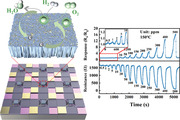

## 1 Introduction

Hydrogen energy has wide applications in the chemical industry, petroleum, nuclear reactors, new energy vehicles, and other fields.^[^ ^1^, ^2^ ^]^ However, H_2_ gas is extremely explosive when the concentrations are higher than 4%.^[^ ^3^ ^]^ Therefore, for secure applications, the development of highly sensitive H_2_ sensors is crucial for on‐line accurate monitoring or leak detection.

Compared with optical or electrochemical gas sensors,^[^ ^4^, ^5^, ^6^, ^7^ ^]^ metal oxide semiconductors (MOS) sensors have the advantages of high sensitivity, all solid state, and low cost.^[^ ^8^, ^9^, ^10^ ^]^ With the development of the Internet of Things and artificial intelligence technology, high‐performance micro‐electro‐mechanical systems (MEMS) sensing chips with low power consumption and small volume have become a research hotspot in recent years.^[^ ^11^, ^12^, ^13^, ^14^ ^]^ For now, MEMS MOS gas sensing chips have been widely fabricated by combining micro hotplate with sensing materials such as ZnO, WO_3_, CuO, and SnO_2_.^[^ ^15^, ^16^, ^17^, ^18^, ^19^, ^20^ ^]^

When the gas sensing chips entered the mass production stage, a series of problems appeared. The preparation of wafer‐level uniform film is an important precondition to achieve high consistency of sensing chips. However, the commonly used methods such as drop‐coating and screen‐printing cannot achieve this goal due to low accuracy and uncontrollable technology.^[^ ^21^, ^22^, ^23^, ^24^ ^]^ At the same time, these methods are easy to cause contact between the sensing materials and the electrode pad, resulting in signal crosstalk between heating electrode and testing electrode, and even affect the effectiveness of the subsequent packaging.^[^ ^25^ ^]^ In addition, noble metal catalysts are very useful to improve gas sensitivity and selectivity.^[^ ^26^, ^27^, ^28^ ^]^ For example, Duan et al.^[^ ^29^ ^]^ established Pd to modulate the kinetic process of gas sensing reaction, and promote the detection ability to H_2_S. Zhang et al.^[^ ^30^ ^]^ deposited Pt catalysts on the SnO_2_ film, which significantly decreased the operating temperature and showed a very high response and a low detection limit toward triethylamine gas. The modification of noble metal is usually prepared on the surface of powder materials by solution impregnation, which is difficult to achieve in wafer‐level manufacturing process.^[^ ^31^ ^]^ In general, it is very important to develop effective gas sensing film pattern deposition methods and noble metal catalytic modification methods at the wafer level for mass manufacturing of sensing chips.^[^ ^12^, ^32^ ^]^

In this work, we developed a simple method combining atomic layer deposition (ALD) and magnetron sputtering to fabricate high‐performance Pd/SnO_2_ film patterns for H_2_ sensing. Based on this method, we can further regulate the grain size and crystallinity of Pd/SnO_2_ by controlling the deposition and annealing processes for enhanced H_2_ sensing performance. The as‐fabricated MEMS H_2_ sensing chips show high consistency, and a wide detection range. The intrinsic sensing enhancement mechanism is further studied based on density functional theory (DFT) calculations.

## 2 Results and Discussion

### 2.1 Preparation and Characterization of Pd/SnO_2_ Film Patterns

**Figure 1:**
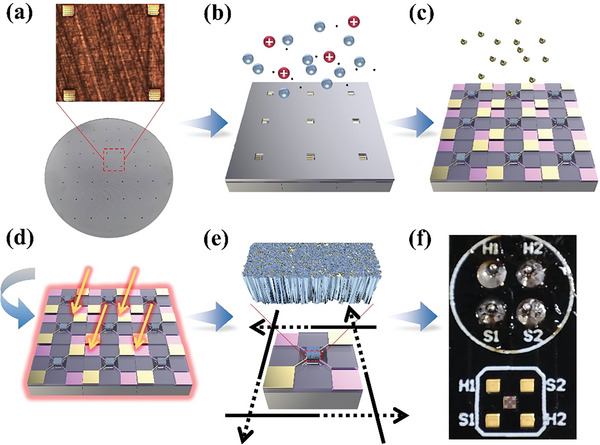
Schematic of the fabrication of Pd/SnO_2_ sensing film patterns and MEMS H_2_ sensing chips: a) The micro hotplate arrays are aligned with the mask, b) SnO_2_ film patterns are deposited in the central sensing area by a mask‐assistant magnetron sputtering method, c) Pd NPs catalysts are further modified on SnO_2_ by ALD, d) the film patterns perform annealing treatment in air‐H_2_‐air, and finally the MEMS H_2_ sensing chips are obtained after e) dicing and f) wire bonding.

**Figure** **1** shows the schematic of the fabrication of Pd/SnO_2_ sensing film patterns and MEMS H_2_ sensing chips. As the substrate of the device, micro hotplate arrays were first fabricated on a wafer through available micromachining techniques, where each micro hotplate consists of a platinum microheater, an insulating layer, and an interdigital test electrode, and the entire suspension structure is supported by an oxide–nitride–oxide layer. The high‐performance Pd/SnO_2_ film pattern is the sensing layer, which is prepared on the surface of micro hotplate arrays by magnetron sputtering deposition, ALD, and subsequent annealing treatment in air‐H_2_‐air atmosphere. After dicing and wire bonding operation, MEMS H_2_ sensing chip was finally prepared. SnO_2_ film patterns samples modified by Pd nanoparticles (NPs) of ALD with 0, 10, 30, and 50 cycle numbers were labeled SnO_2_, 10Pd/SnO_2_, 30Pd/SnO_2_, and 50Pd/SnO_2_, respectively.

**Figure 2:**
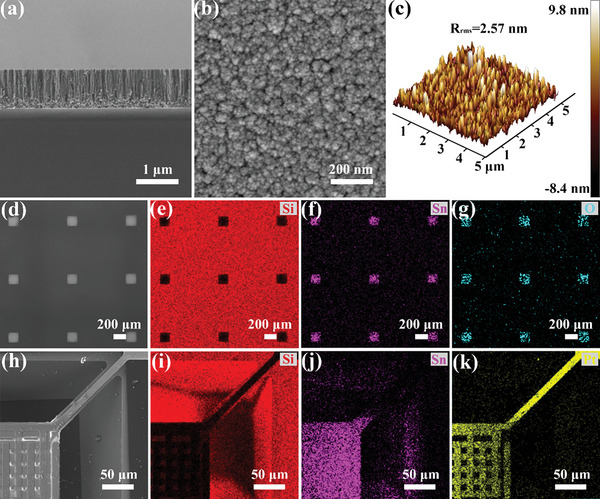
a,b) The cross‐section and  top view SEM images, and c) AFM images of SnO_2_ film patterns. SEM and corresponding EDS images of the 150 µm×150 µm square SnO_2_ film patterns prepared on d–g) silicon substrate and h–k) micro hotplate.

It is well known that the consistency of sensing film is quite crucial to the mass manufacturing of sensing chips.^[^ ^33^ ^]^ As the key to determining the gas sensing response of the sensing chip, the consistency of SnO_2_ film patterns was first studied. The pure SnO_2_ film patterns on silicon wafer were prepared by mask‐assistant deposition method, and scanning electron microscopy (SEM) and atomic force microscopy (AFM) images in **Figure** **2**a–c and Figure S1, Supporting Information, show that the as‐prepared film patterns are composed of uniformly distributed smaller particles with a measured surface root mean square roughness (*R*_rms_) of 2.57 nm. The film thicknesses were measured for five representative areas throughout the whole wafer and the values are 971.4, 963.9, 960.2, 952.7, and 945.3 nm. Besides, X‐ray Diffraction (XRD) patterns of SnO_2_ films (Figure S2, Supporting Information) prepared three times show that the as‐prepared films are pure stoichiometric SnO_2_ with the tetragonal structure (JCPDS No. 41–1445), and such films have similar grain sizes, that is, 4.567, 4.767, and 4.433 nm which were calculated based on the Scherrer equation, and the relative standard deviation (RSD) value is 3.66%. The results indicate that the as‐prepared SnO_2_ film patterns have good consistency.

A high‐quality patterning of the gas sensing films in the central sensing areas of the micro hotplates ensures full exposure of the test electrode and heating electrode pads, and avoids signal crosstalk caused by the over‐coverage of sensing film, which is the basis for chip packaging and testing.^[^ ^25^ ^]^ We prepared 150 µm×150 µm square SnO_2_ film patterns on a silicon wafer to evaluate the as‐used method. As shown in Figure 2d, the square film patterns are well deposited on the substrate without any deformation and the edges are very smooth. The energy‐dispersive spectroscopy (EDS) pattern of SnO_2_ film pattern is shown in Figure 2e–g. It can be clearly seen that the elements in the central areas are Sn and O, while the element in the shielded areas is Si from the substrate. There is an obvious difference between these two areas. The results demonstrate that the mask‐assistant deposition method is very useful to prepare film patterns. Similarly, Figure 2h–k shows that the deposition areas of a component element Sn in the film are the same as that of Pt micro heating electrodes. The result confirms that the SnO_2_ film patterns are accurately deposited in the central sensing areas above MEMS micro hotplate arrays.

**Figure 3:**
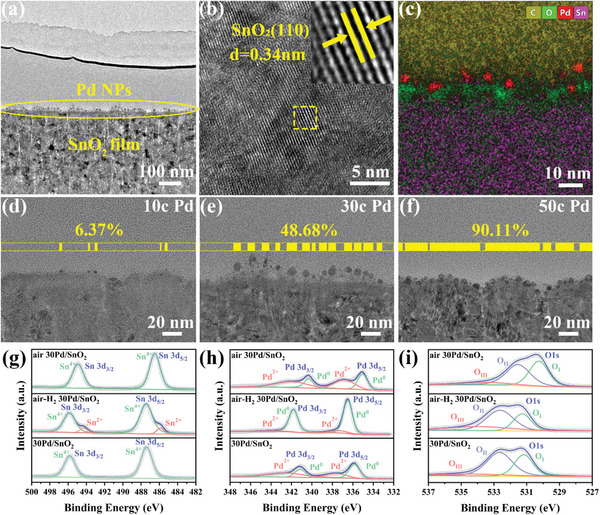
a) The cross‐section TEM image, b) HR‐TEM image, and c) the elemental mapping image of the 30Pd/SnO_2_ sample. d–f) The Pd NPs’ size and density of 10, 30, and 50Pd/SnO_2_ samples. High‐resolution XPS spectra of air 30Pd/SnO_2_, air‐H_2_ 30Pd/SnO_2_, and 30Pd/SnO_2_: g) the Sn 3d region, h) the Pd 3d region, and i) the O 1s region.

Additionally, the crystallinity of the semiconductor film as well as the density and particle size of the catalyst have important influences on sensing performance.^[^ ^29^, ^34^, ^35^ ^]^ The cross‐section transmission electron microscope (TEM) images of 30Pd/SnO_2_ showed in **Figure** **3**a,b reveal that the film patterns possess a polycrystalline structure, and the interlayer distance is measured to be about 0.34 nm corresponding to the (110) plane of SnO_2_. Moreover, Pd catalysts are distributed on the surface of SnO_2_ films in the form of nanoparticles, which are demonstrated by the element mapping patterns (Figure 3c). To compare the particle size and density of Pd NPs at different ALD cycle numbers, we calculated the proportion of Pd NPs based on the same length scale. As shown in Figure 3d–f, Figures S3 and S4, Supporting Information, as the cycle numbers vary from 10 to 30, the particle size changes from ≈3 to ≈6 nm, and when the cycle number continues to increase, the particle size remains unchanged. The density of Pd nanoparticles can depend on the ratio of the vertical projection length of Pd nanoparticles to the fixed entire length, shown in Figure 3d–f. The results show that the corresponding ratio values are 6.37%, 48.68%, and 90.11% for 10, 30, and 50Pd/SnO_2_ samples, respectively, indicating that the density gradually increases with the increase of the cycle numbers, which is closely related to the gas sensing performance.

To further study the structure of film patterns, XRD (Figure S5, Supporting Information) and X‐ray photoelectron spectroscopy (XPS) (Figure 3g–i) are applied. The XPS measurements of air 30Pd/SnO_2_, air‐H_2_ 30Pd/SnO_2_, and 30Pd/SnO_2_ were performed to further characterize the surface chemical states and the electron interactions. Figure 3g shows that the two peaks of air 30Pd/SnO_2_ at 486.5 and 494.9 eV are identified as Sn 3d_5/2_ and Sn 3d_3/2_, respectively, indicating that Sn presents Sn^4+^. After annealing in a H_2_ atmosphere, a mixed valence state of Sn^4+^ and Sn^2+^ appears and the peak value of Sn^4+^ shifts 0.95 eV toward a higher binding energy. Notably, the peak value of Sn^4+^ does not change after the final air annealing even though Sn^2+^ disappears. The peaks of Pd 3d in Figure 3h exhibit the existence of Pd^0^ and Pd^2+^ in air‐H_2_ 30Pd/SnO_2_, air 30Pd/SnO_2_, and 30Pd/SnO_2_. However, the content of Pd^0^ in 30Pd/SnO_2_ is significantly higher than that in air 30Pd/SnO_2_, and the fitting peak values all shift 0.8 eV. The O 1s spectra (Figure 3i) show similar peak shift with the change of annealing treatment, and the peaks named O_I_, O_II_, and O_III_ correspond to lattice oxygen, oxygen vacancy, and surface‐adsorbed oxygen, respectively.^[^ ^36^ ^]^ Differently, the peaks of Sn and O in pure SnO_2_ (Figure S6, Supporting Information) remain unchanged under different annealing treatments. Therefore, it is believed that the shift of peaks in 30Pd/SnO_2_ is attributed to the electron interaction, which is driven by the different electronegativity of the Sn and Pd metal ions.

### 2.2 Pd/SnO_2_ Film Patterns Applied for Controllable MEMS Sensing Chips

**Figure 4:**
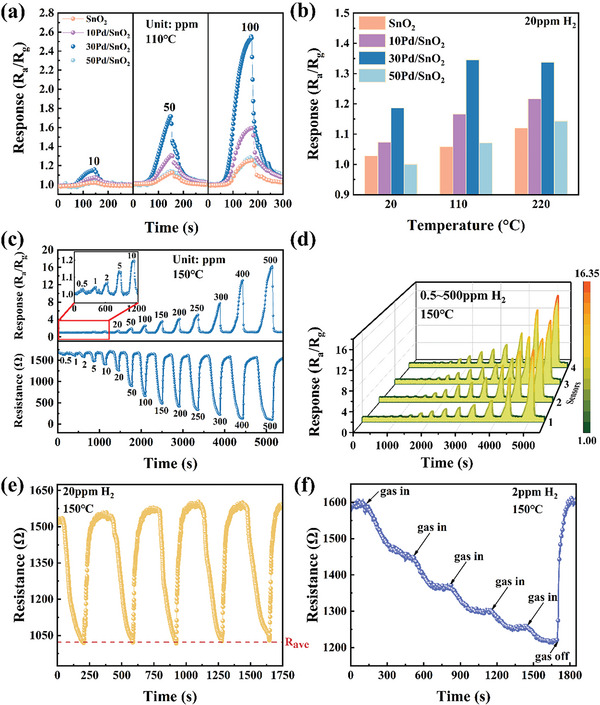
a) The gas response of Pd/SnO_2_ sensing chips with different Pd content toward 10/50/100 ppm H_2_ at 110 °C and b) toward 20 ppm H_2_ at 20/110/220 °C. c) Dynamic sensing behavior of the sensing chips toward H_2_ with a concentration range of 0.5–500 ppm at 150 °C. d) The response consistency of 30Pd/SnO_2_ sensing chips from four different wafer regions. e) The repeatability test toward 20 ppm H_2_ at 150 °C. f) Experimental results of five continuous injections without recovery.

To evaluate H_2_ sensing performance of the regulated Pd/SnO_2_ film patterns, a series of measurements were performed. Primarily, we compared the responses of 30Pd/SnO_2_ and air 30Pd/SnO_2_ samples toward 10, 50, and 100 ppm H_2_. As shown in Figure S7a, Supporting Information, the former shows higher response at all H_2_ concentrations and the same conclusion can be obtained under the operating temperatures ranging from room temperature to 220 °C, as shown in Figure S7b, Supporting Information. It proves that H_2_ and air cycle annealing steps are useful for the enhancement of response. As shown in **Figure** **4**a,b, when the number of cycles is less than 30, the response would increase with increasing the content of Pd (30Pd/SnO_2_ > 10Pd/SnO_2_ > SnO_2_), and this conclusion is valid at room temperature to 220 °C. However, when the cycles reach 50, the response decreases significantly and approaches that of pure SnO_2_. This interesting phenomenon attracted our attention. Since SnO_2_ is very surface‐sensitive material, we prepared SnO_2_ sensing chips three times under the same conditions in order to evaluate the consistency of SnO_2_ films. As shown in Figure S8, Supporting Information, Pd/SnO_2_ films have almost the same response to H_2_ gas and the RSD value of the gas response to 100 ppm H_2_ is as low as 1.68%. Therefore, it is thought that the Pd/SnO_2_ film patterns are consistent and the reduced response of 50Pd/SnO_2_ is mainly attributed to large coverage of Pd NPs on the SnO_2_ film surface (Figure 3f). Normally, such a small amount of Pd does not lead to a reversal of the increasing trend of response, which is different from the results of Pd catalysts prepared by chemical methods such as impregnation modification. Possible causes are discussed in the sensing mechanism part.

The above results show that 30Pd/SnO_2_ film pattern exhibits the best H_2_ sensing performance, so we adopted it to the MEMS sensing chips. The optimum operating temperature of 30Pd/SnO_2_ is 150 °C (Figure S9, Supporting Information), and the resistance and response value of the sensing chips are obtained at this temperature (Figure 4c). With the increased concentration of H_2_, the resistance and the response vary gradually with linearity (Figure S10, Supporting Information). And, at all H_2_ concentrations, even up to 500 ppm, the resistance changes immediately as soon as injecting H_2_. For recovery time, 70% of the resistance change value can be recovered within 30 s, and 90% of the resistance change value takes a long time (92 s) to recover. For the detection limit, when H_2_ concentration is as low as 0.5 ppm, there is still a noticeable change in resistance and response value (signal‐to‐noise ratio > 3). This sensing chip shows a lower detection limit and a wider detection range up to three orders of magnitude compared with some previously reported H_2_ sensors (Table S1, Supporting Information). As we know, the consistency of the gas sensing performances of the same batch of chips is key to wafer‐level production. Therefore, we tested the responses of four sensors from different locations on the wafer at the same operating temperature (150 °C) and the same concentration of H_2_ (0.5–500 ppm). As can be seen from the Figure 4d and Figure S11, Supporting Information, four sensors show similar response with almost zero error at 0.5–100 H_2_ concentration, and 8.7% error (less than ±10%) at the high H_2_ concentration of 500 ppm.

Furthermore, five gas injection/extraction cycles of 20 ppm H_2_ are carried out to evaluate the repeatability of 30Pd/SnO_2_, which is vital for the detection accuracy in unknown atmospheres. As shown in Figure 4e, the resistance changes in the range of 1020–1029 Ohms when exposed to H_2_ gas, and the fluctuation of the value is much smaller than that of the base value. Further, we performed five consecutive injections of H_2_ at a low concentration of 2 ppm to evaluate the resolution. Figure 4f shows that with each injection of H_2_, the resistance decreases significantly, and then remains stable after a certain response time, until the test chamber is opened, the resistance eventually increases. The results indicate that the H_2_ sensing chips have good repeatability and resolution.

### 2.3 H_2_ Sensing Mechanism

**Figure 5:**
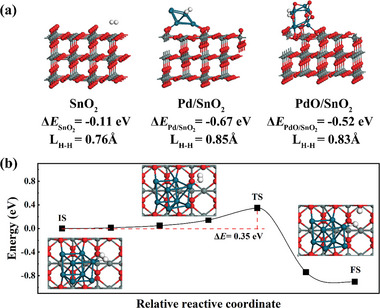
a) The adsorption energy and H—H bond length of H_2_ adsorbed on the surfaces of SnO_2_, Pd/SnO_2_, and PdO/SnO_2_. b) The intermediate structures and the transition state energies in H_2_ dissociated into two H species on the surfaces of Pd/SnO_2_ (The atoms are displayed in different colors: Sn atoms in grey, O atoms in red, Pd atoms in blue, and H atoms in white, respectively).

To understand the gas sensing enhancement mechanism, the DFT calculations were performed to study the adsorption and reaction of H_2_ molecules on Pd/SnO_2_ surface. As shown in **Figure** **5**a, the models of H_2_ absorbed on the surface of SnO_2_, Pd/SnO_2_, and PdO/SnO_2_ were constructed to calculate the adsorption energy and H—H bond length, respectively. Among them, Pd/SnO_2_ model has the lowest adsorption energy for H_2_, with a value of −0.67 eV, which means H_2_ is easily adsorbed on it. Meanwhile, the H—H bond lengths for the SnO_2_, Pd/SnO_2_, and PdO/SnO_2_ models are about 0.76, 0.85, and 0.83 Å, respectively, which further prove that Pd novel metal has the best catalytic performance than other catalysts and can effectively promote H_2_ dissociation. In addition, we also calculated the energy of the transition‐state structure, as shown in Figure 5b. It is observed that the dissociation of H_2_ molecules into two H species on the surfaces of Pd/SnO_2_ only needs to overcome the low energy barrier of 0.35 eV. Two H species bind with Pd atom and O atom, respectively, and the forming structure has a lower energy of −1.0 eV than the transition‐state structures. Similarly, the reaction path of H_2_ dissociation on the SnO_2_ surface were also simulated and calculated. Compared with Pd/SnO_2_ surface, the energy barrier of H_2_ dissociation at SnO_2_ surface increases to 1.13 eV, which proves that Pd catalyst plays the important role of active sites, and it has an excellent catalytic behavior of H_2_ sensing than pure SnO_2_ material (Figures S12 and S13, Supporting Information).

**Figure 6:**
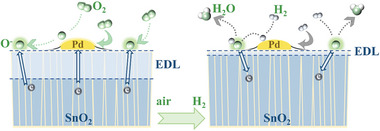
Schematic of H_2_ gas sensing mechanism of 30Pd/SnO_2_.

Based on the calculation and experiment results, the gas sensing enhancement mechanism was then proposed (**Figure** **6**). When pure SnO_2_ is exposed in air, O_2_ can be adsorbed on SnO_2_ and these oxygens capture free electrons from the conduction band of SnO_2_ to form O^−^, O_2_^−^, or O^2−^, which is thermally activated and closely depends on the operating temperature.^[^ ^37^, ^38^ ^]^ In this process, an electron depletion layer (EDL) is formed, and the resistance of the sensing chip increases. As an effective catalyst, a small amount of Pd can promote the adsorption and dissociation processes of O_2_ based on “oxygen spillover” and “back‐spillover” effects, which help O_2_ capture more electrons from SnO_2_ and result in a wider EDL. Besides, the Pd/SnO_2_ Schottky barrier and the strong electron acceptor PdO would cause SnO_2_ to lose more electrons.^[^ ^39^, ^40^ ^]^ However, based on the HR‐TEM results in Figure 3f, an excessive Pd NPs of 50 cycle numbers modified on the SnO_2_ surface may hinder the contact between O_2_ and SnO_2_, thereby reducing the EDL width and negatively affecting the gas sensitivity response.

When the chips are exposed to H_2_, as shown in the right part of Figure 6, H_2_ molecules are adsorbed and decomposed into H atoms on the surface of SnO_2_.^[^ ^41^ ^]^ The obtained hydrogen atoms mainly react with oxygen atoms to form surface hydroxyl groups.^[^ ^42^ ^]^ The hydroxyl group has low electron affinity, it releases electrons back to the SnO_2_ and desorbs above 100 °C, which significantly decreases the EDL width and resistance.^[^ ^39^ ^]^ The reactions can be expressed as follows.

(1) $$H_{2\left(\mathrm{ads}\right)}\to 2H_{\left(\mathrm{ads}\right)}$$

(2) $$H_{\left(\mathrm{ads}\right)}+O_{\left(\mathrm{ads}\right)}^{-}\to {\mathrm{OH}}_{\left(\mathrm{ads}\right)}^{-}$$

(3) $${\mathrm{OH}}_{\left(\mathrm{ads}\right)}^{-}+H_{\left(\mathrm{ads}\right)}\to H_{2}O_{\left(g\right)}+e^{-}$$

The calculation results indicate that modification of a small amount of Pd catalyst promotes the adsorption of H_2_ because of the low binding energy.^[^ ^43^, ^44^ ^]^ The “hydrogen spillover” and “back‐spillover” effects further improve the adsorption and dissociation ability of H_2_. Meanwhile, the spillover H species also help to decrease the reduction temperature of active oxygen species, which further promotes the return of more electrons to SnO_2_. In addition, H_2_ can reduce PdO to Pd, result in the disappearance of electron interactions,^[^ ^45^ ^]^ and even reduce to the low‐work function PdH*_x_*, which promotes the reverse transfer of electrons.^[^ ^46^ ^]^ Above all, the return of a large number of electrons results in a decrease in a narrowing of the EDL and the resistance of the chips.

## 3 Conclusion

This work reported a simple method combining ALD and magnetron sputtering to fabricate high‐performance Pd/SnO_2_ film patterns applied for gas sensing chips. SnO_2_ film was accurately deposited in the central areas of MEMS micro hotplate arrays by a mask‐assistant method, and the patterns show good consistency in thickness on the whole wafer. To further obtain the optimal crystallinity and grain size for H_2_ sensing, deposition of Pd by 30 cycles of ALD method and air‐H_2_‐air annealing process are finally performed. The as‐prepared MEMS sensing chips show many advantages, such as a wide detection range from 0.5 to 500 ppm, high resolution, and good repeatability. The DFT calculations demonstrate that an appropriate amount of Pd NPs modified on the surface of support has better adsorption ability and dissociation effect for H_2_. This work will provide a general method for high consistency and controllable fabrication of high‐end gas sensing chips in the future.

## 4 Experimental Section

### Preparation of SnO_2_ Film Patterns

The micro hotplate arrays were aligned and locked together with the mask to ensure that there was no displacement in any direction during transfer and deposition. SnO_2_ film patterns were then deposited in the central sensing areas of the micro hotplate arrays by DC magnetron sputtering method. A 99.99% high‐purity metal Sn target was assembled in the chamber and the chamber was evacuated to a pressure to <5×10^−4^ Pa before beginning the deposition process. Under these conditions, a 17 min sputtering process was performed using 100 W DC power with a gas mixture of 24 sccm Ar and 8 sccm O_2_ at a pressure of 1.0 Pa. The substrates were kept at room temperature in the deposition process.

### Modification of Pd Nanoparticles

Pd catalysts were modified on the surface of SnO_2_ film patterns by ALD technology. In this process, the reaction chamber temperature was 200 °C, and the pipe temperature was 150 °C. Primarily, a 10 ms high‐purity N_2_ pulse was introduced into the Pd(hafc)_2_ precursor source bottle to form a large pressure. Subsequently, due to the pressure difference, the trace gaseous Pd(hafc)_2_ precursor source was fed into the reaction chamber for a pulse time of 1000 ms. The chamber was then purged with high‐purity N_2_ for 25 s, followed by the introduction of another precursor, tBuNHNH_2_, with a pulse time of 200 ms. The above process was a complete cycle of Pd deposition, and to explore the optimum content, 10, 30, and 50 cycles of Pd deposition were performed.

### Annealing of Pd/SnO_2_ Film Patterns

Annealing processes under different atmospheres were performed to regulate the grain size and crystallinity of Pd/SnO_2_ film patterns for improving the sensitivity of chips. First, the chips were annealed in an air atmosphere at 400 °C for 2.5 h, and the heating rate was 5 °C s^−1^; then, were annealed in hydrogen atmosphere at 250 °C for 2.5 h at a rate of 20 °C min^−1^. Last, the air annealing treatment was repeated according to the first step.

### Material Characterization

AFM (Bruker Dimension EDGE) was applied to measure the surface roughness and obtain the surface information. Field emission SEM (Gemini SEM 300) was used to characterize the morphology and thickness of SnO_2_ film in different regions of the wafer. The fine structure of Pd/SnO_2_ thin film was observed by TEM and HR‐TEM (Thermo Scientific Talos F200X). High Angle ring dark field scanning transmission electron microscopy and energy dispersive X‐ray spectroscopy were employed to analyze the elements and their distributions, especially the Pd element. XRD (Shimadzu Corporation XRD‐7000) and XPS (AXIS‐ULTRA DLD‐600 W) were used to study the phase structure, chemical composition, and valence states of samples.

### Gas Sensing Measurements

H_2_ sensing chips were placed in a test chamber with good sealing performance to keep the internal ambient temperature and humidity unchanged. In the test process, a heating voltage is applied to the heater of the MEMS gas sensing chips through a voltage source, which were pre‐calibrated using an infrared thermal camera equipment (Fluke RSE60). The measurement system and infrared images are shown in Figures S14 and S15, Supporting Information. The resistance of sensing chips was obtained by digital multimeters (Agilent U3606A). Initially, the baseline of the sensor resistance remained stable in air. The target gas was then injected and the resistance was varied until it stabilized again. The response of the sensing chip (*S* = *R*_a_/*R*_g_) was calculated according to the ratio value of the resistance in air (*R*_a_) and resistance in target gas (*R*_g_).

### Computational Methods

The DFT calculation was carried out by using the Vienna ab initio simulation package code. The Pd/SnO_2_ and PdO/SnO_2_ structures were built through setting the (111) of Pd and (101) of PdO on the optimized (110) surface of SnO_2_. The cutoff energy was set to 400 eV and the k‐point grid was taken as 2 × 2 × 1. The convergence values of force and energy were about 0.05 eV Å^−1^ and 1.0 × 10^−5^ eV per atom. The adsorption energy (Δ*E*_ads_) of SnO_2_, Pd/SnO_2_, and PdO/SnO_2_ were calculated by the following formula: Δ*E*_ads_ = *E*_adsorbate/slab_ − (*E*_slab_ + *E*_adsorbate_), where *E*_adsorbate/slab_ is the total energy of the materials and target gas molecules, *E*_slab_ is the energy of the materials, and *E*_adsorbate_ is the energy of isolate gas molecules. Furthermore, the final H—H bond length results were obtained. In addition, the adsorption and dissociation processes of H_2_ molecules were studied by the climbing‐image nudged elastic band method.

## Conflict of Interest

The authors declare no conflict of interest.

## Supporting information

Supporting Information

## Data Availability

The data that support the findings of this study are available from the corresponding author upon reasonable request.
