# Supplementary material for: Wafer‐Level Manufacturing of MEMS H2 Sensing Chips Based on Pd Nanoparticles Modified SnO2 Film Patterns
Source: Adv Sci (Weinh). 2023 Jul 3;10(26):2302614. doi: 10.1002/advs.202302614 (PMC10502828; doi:10.1002/advs.202302614)
Supplement: Supplementary file 1 — Supporting Information [file ADVS-10-2302614-s001.pdf]

ADVANCED SCIENCE
Open Access

Supporting Information

for *Adv. Sci.*, DOI 10.1002/advs.202302614

Wafer-Level Manufacturing of MEMS H_2 Sensing Chips Based on Pd Nanoparticles Modified SnO_2 Film Patterns

*Zheng Zhang, Liyang Luo, Yanlin Zhang, Guoliang Lv, Yuanyuan Luo and Guotao Duan**

Supporting Information

Wafer-level manufacturing of MEMS H_2 sensing chips based on Pd nanoparticles modified SnO_2 film patterns

Zheng Zhang [a, §], Liyang Luo [a, §], Yanlin Zhang [a], Guoliang Lv [a], Yuanyuan Luo [b], Guotao Duan [a, c, *]

[a] School of Integrated Circuits, Huazhong University of Science and Technology, Wuhan 430074, China

[b] Key Laboratory of Materials Physics, Institute of Solid State Physics, HFIPS, Chinese Academy of Sciences, Hefei 230031, China

[c] Wuhan National Laboratory for Optoelectronics, Huazhong University of Science and Technology, Wuhan 430074, China

[§] These authors contributed equally to this work.

[*] Corresponding author: duangt@hust.edu.cn

Table S1 Comparison of sensing performances for various H_2 sensors

Sensing materials	Substrate	Methods	Concentration (ppm)	Temperature (°C)	Ref.
Pd/SnO_2 spheres	alumina	one-pot hydrothermal	500~1000	330	[1]
Pd@Pt/SnO_2 hybrids	ceramic	hydrothermal	100~1000	25	[2]
PtSnx-rGO-SnO_2 nanocomposites	ceramic	refluxing reaction	1~1000	175	[3]
Pd/SnS_2/SnO_2 nanocomposites	ceramic	hydrothermal	100~10000	300	[4]
Pd NPs/SnO_2 NW	silicon	SnO_2: vapor-liquid-solid<br>Pd: UV irradiation	1~400	300	[5]
Pd NPs/SnO_2	silicon	SnO_2: magnetron sputtering<br>Pd: ALD	0.5~500	150	This work

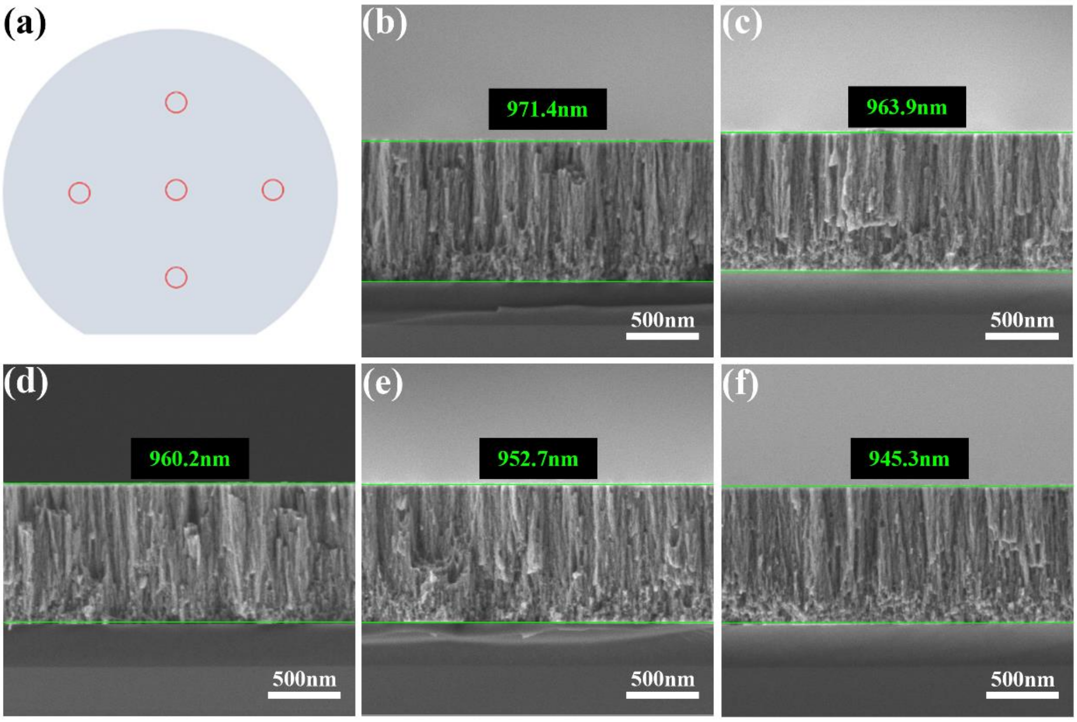

Figure S1. The cross-section SEM images of SnO_2 film in five representative areas.

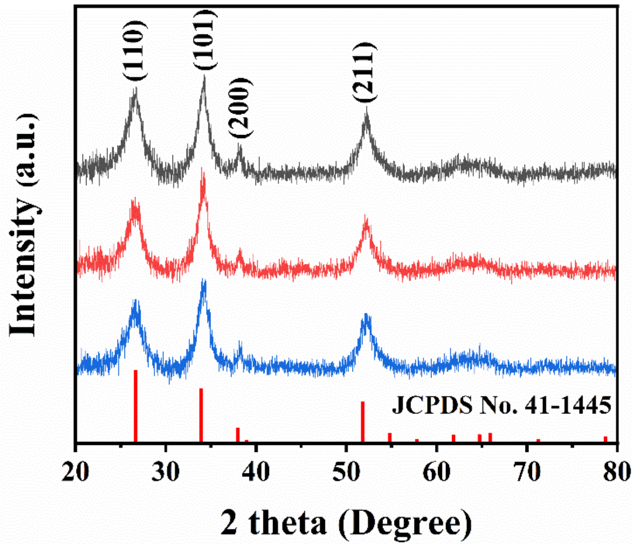

Figure S2. XRD patterns of pure SnO_2 films.

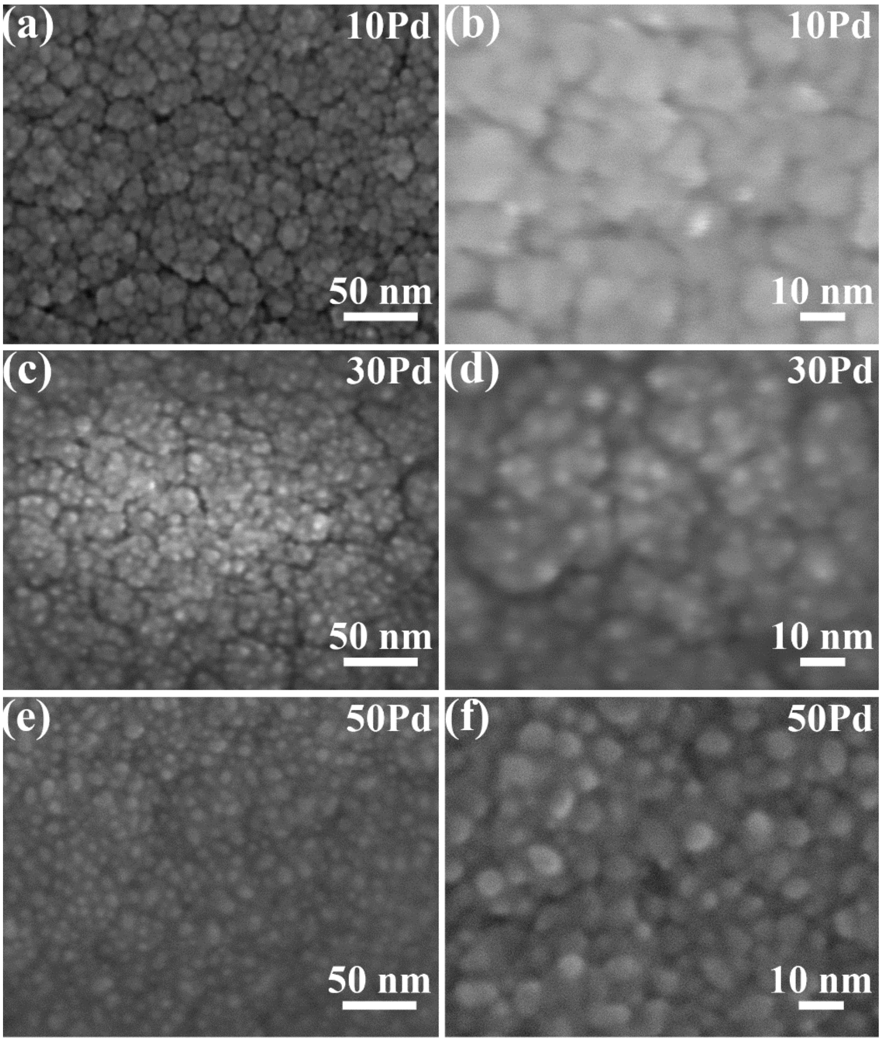

Figure S3. HR-SEM images of (a)-(b) 10, (c)-(d) 30, and (e)-(f) 50Pd/SnO_2 samples.

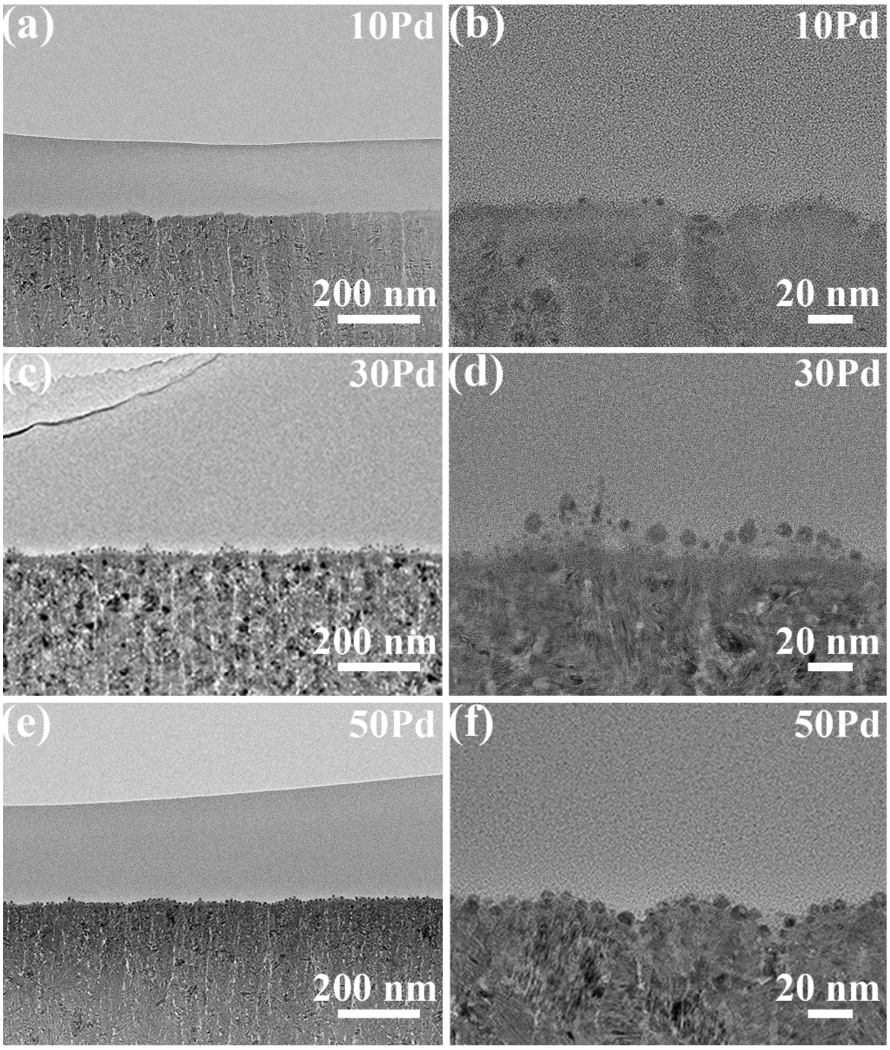

Figure S4. HR-TEM images of (a)-(b) 10, (c)-(d) 30, and (e)-(f) 50Pd/SnO_2 samples.

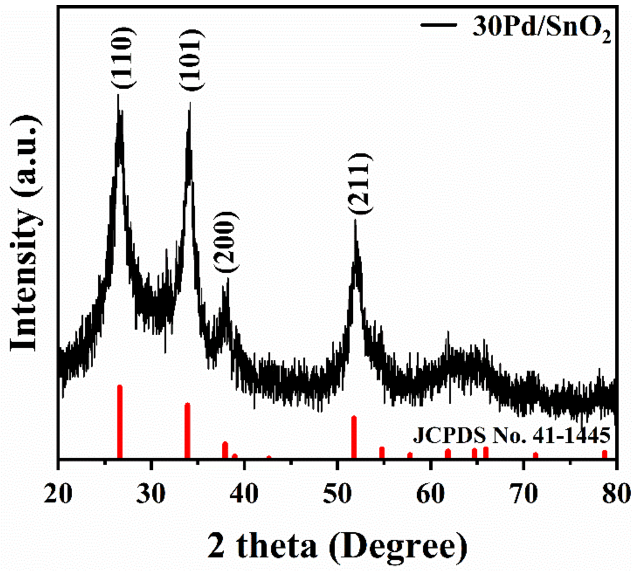

Figure S5. XRD pattern of 30Pd/SnO_2 sample.

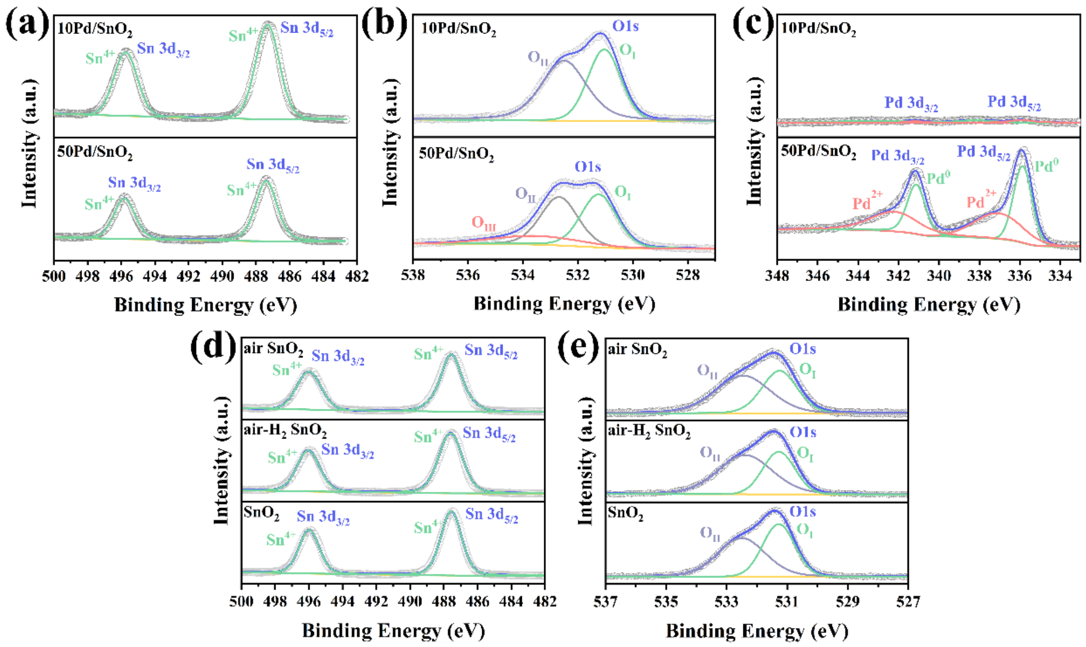

Figure S6. High-resolution XPS spectra of 10Pd/SnO_2 and 50Pd/SnO_2: a) the Sn 3d region, b) the Pd 3d region and c) the O 1s region. High-resolution XPS spectra of pure SnO_2 with different annealing treatment: d) the Sn 3d region and e) the O 1s region.

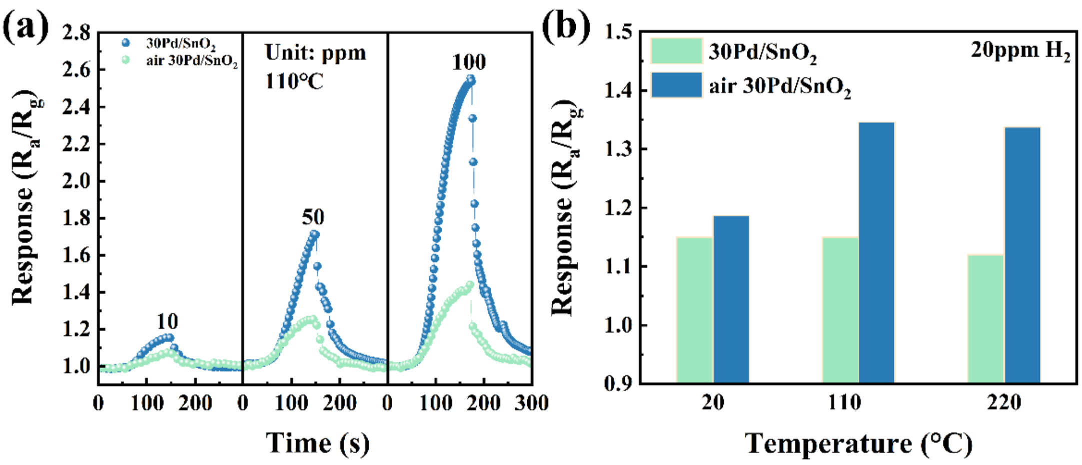

Figure S7. a) The gas response of Pd/SnO_2 sensing chip with different calcination processes for 10, 50, and 100 ppm H_2 at 110 °C and b) for 20 ppm H_2 at 20, 110, and 220 °C.

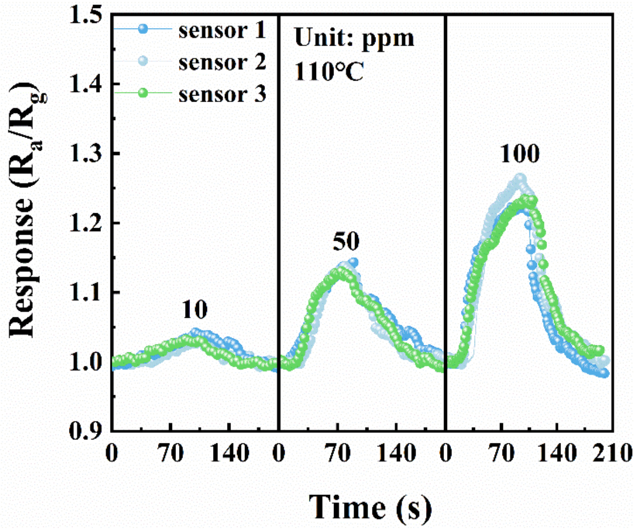

Figure S8. The response of pure SnO_2 sensing chips towards 10, 50, and 100 ppm H_2 with the same preparation conditions.

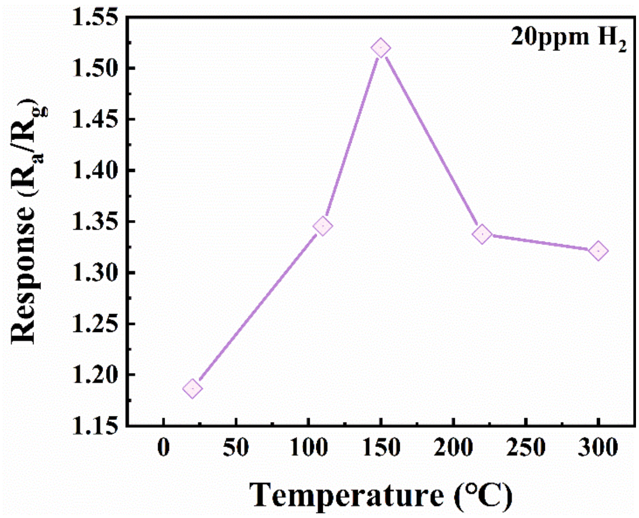

Figure S9. Response comparison of 30Pd/SnO_2 sensing chip at different operating temperatures.

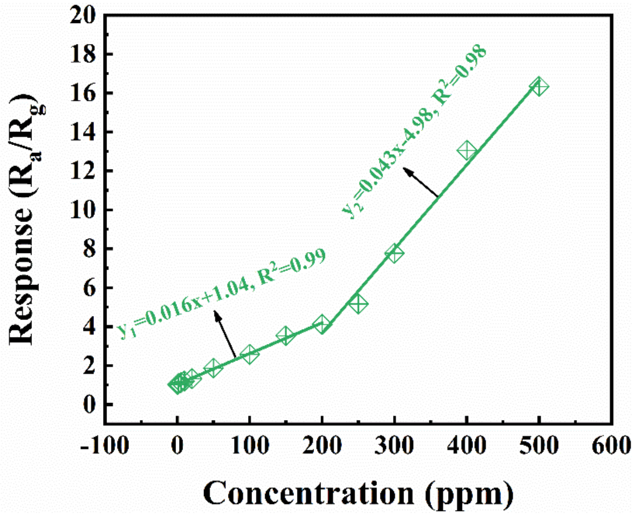

Figure S10. The corresponding linear fitting results between gas response and gas concentration.

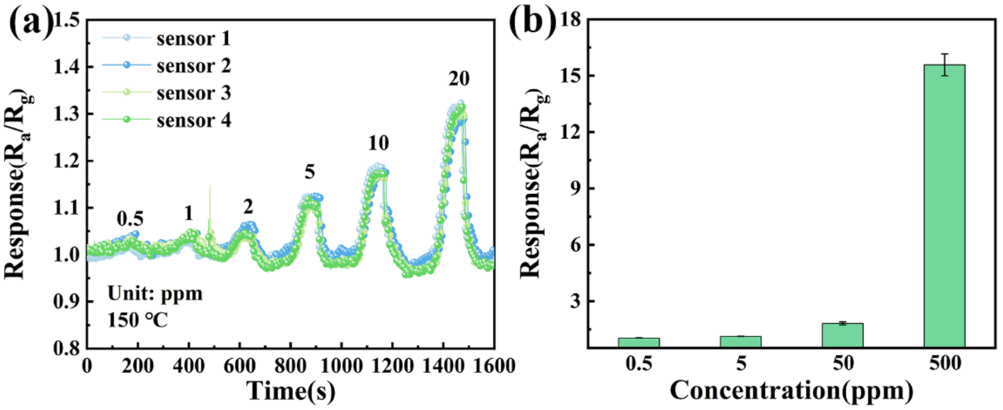

Figure S11. The response consistency and error bar of $30Pd/SnO_2$ sensing chips from 4 different wafer regions.

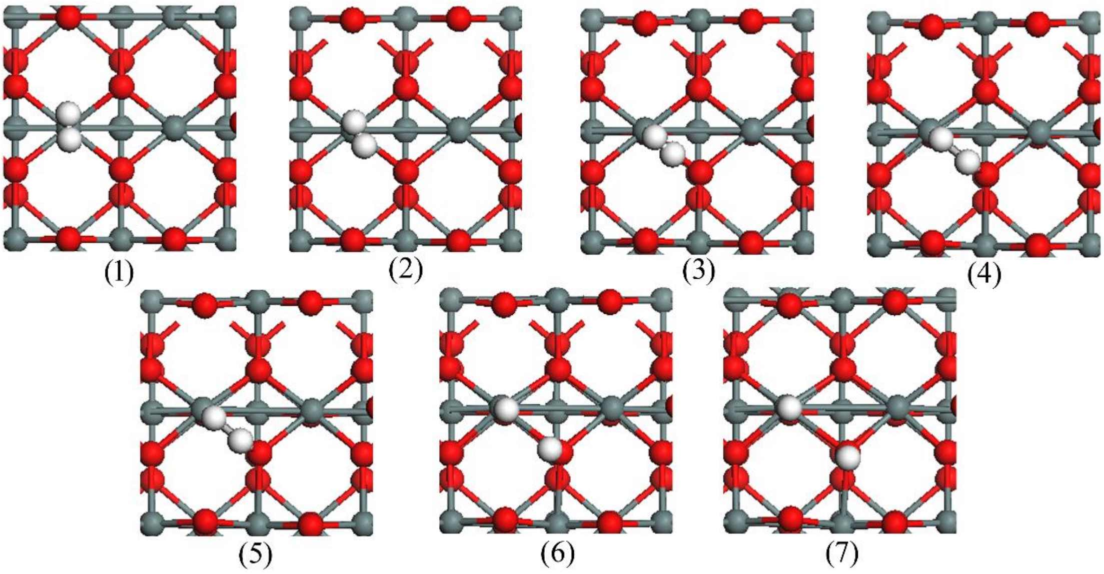

Figure S12. The intermediate structures of H_2 dissociation into two H species on the surface of SnO_2.

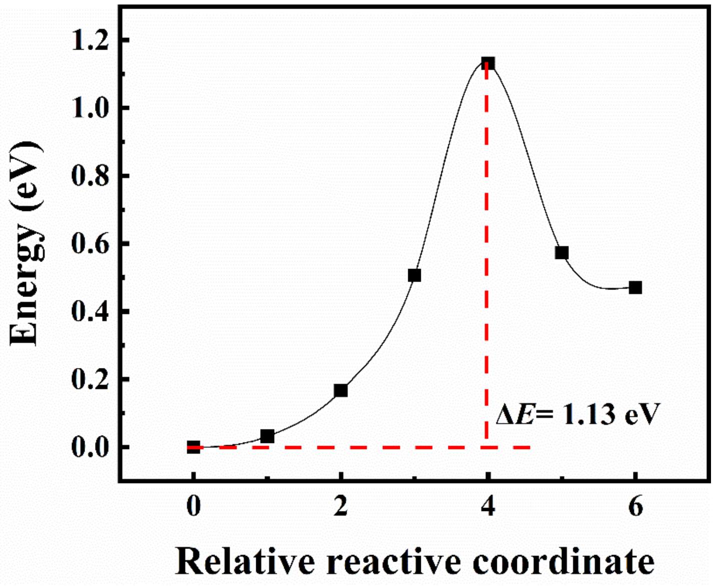

Figure S13. The transition state energies of H_2 dissociation into two H species on the surface of SnO_2.

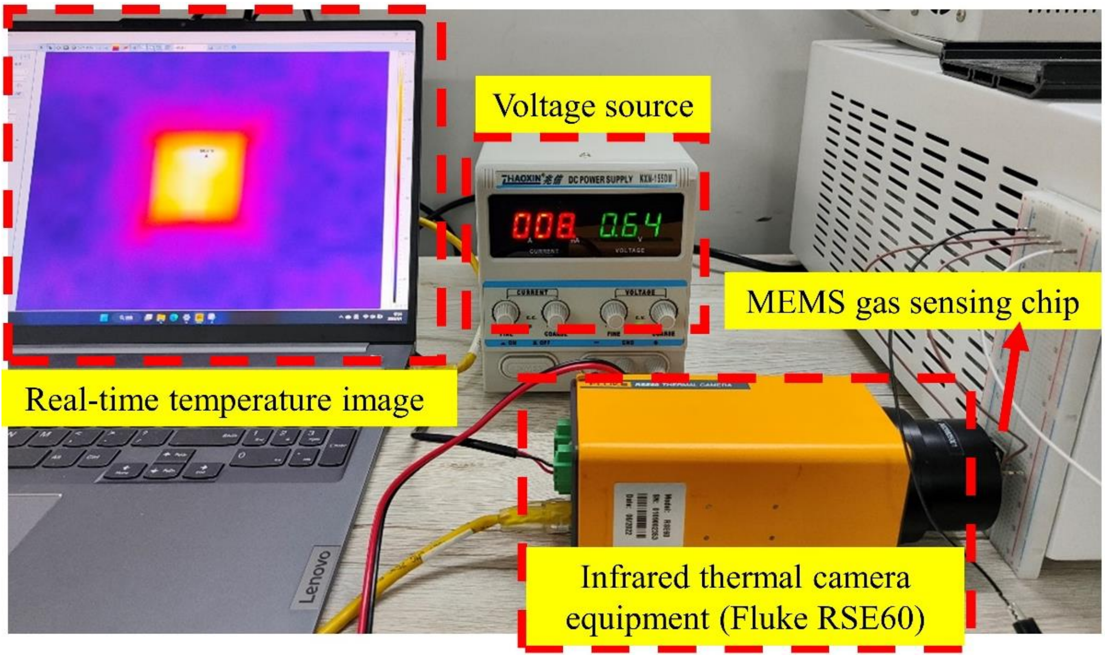

Figure S14. The infrared temperature measurement system.

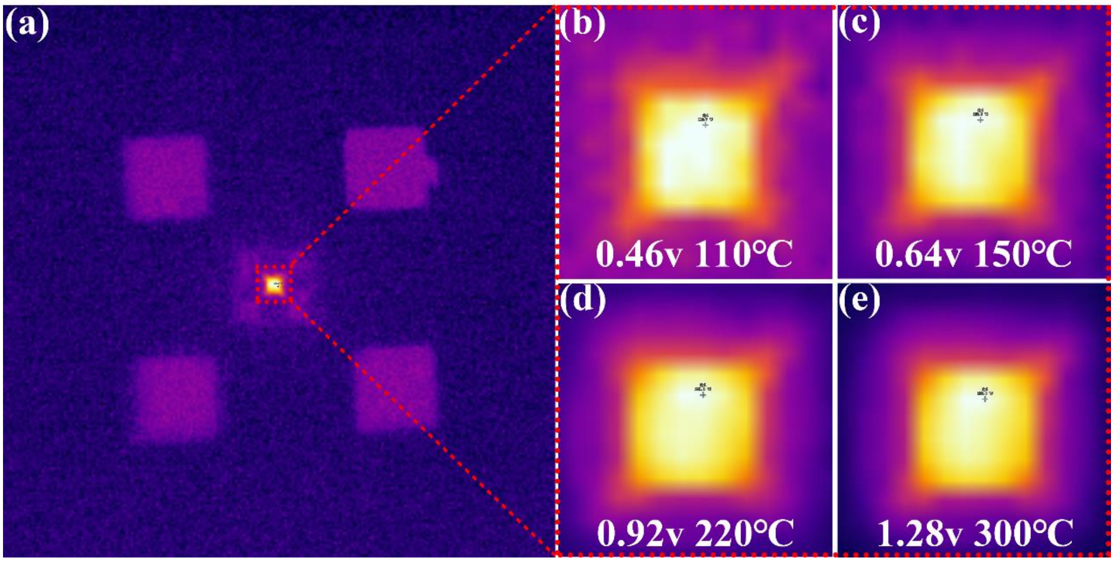

Figure S15. The infrared images of the chips under different applied voltages.

References

[1] P. Duan, H. Xiao, Z. Wang, Q. Peng, K. Jin, J. Sun, *Sensors and Actuators B: Chemical* **2021**, *346*, 130557.

[2] X. Meng, M. Bi, W. Gao, *Sensors and Actuators B: Chemical* **2022**, *370*, 132406.

[3] G. Li, Y. Shen, S. Zhao, A. Li, S. Gao, D. Wei, Z. Yuan, F. Meng, D. Meng, *Sensors and Actuators B: Chemical* **2022**, *368*, 132146.

[4] X. Meng, M. Bi, Q. Xiao, W. Gao, *Sensors and Actuators B: Chemical* **2022**, *359*, 131612.

[5] Z. Cai, S. Park, *Sensors and Actuators B: Chemical* **2020**, *322*, 128651.
